# Numerical modeling and analysis of coaxial electrohydrodynamic jet printing

**DOI:** 10.1038/s41598-022-05596-y

**Published:** 2022-02-04

**Authors:** Dazhi Wang, Zeshan Abbas, Liangkun Lu, Xiangyu Zhao, Pengfei Xu, Kuipeng Zhao, Penghe Yin, Junsheng Liang

**Affiliations:** 1 Key Laboratory for Micro/Nano Technology and System of Liaoning Province, Dalian University of Technology, Dalian, 116024 China; 2 Ningbo Institute of Dalian University of Technology, Ningbo, 315000 China; 3 Key Laboratory for Precision and Non-Traditional Machining Technology of Ministry of Education, Dalian University of Technology, Dalian, 116024 China

**Keywords:** Engineering, Nanoscience and technology

## Abstract

Coaxial electrohydrodynamic jet (CE-Jet) printing is an encouraging method for fabrication of high-resolution micro and nanostructures in MEMS systems. This paper presents a novel simulation work based on phase field method which is considered as a precise technique in fluid dynamics. The study explores influence of various parameters such as applied voltage, needle-substrate distance, dynamic viscosity, relative permittivity, needle size and flow rate on stability and resolution of CE-Jet morphologies. The morphology of CE-Jet exhibits that width of cone-jet profile and printed structures on substrate were directly proportional to relative permittivity and flow rate. In addition, it was inversely proportional to dynamic viscosity and applied voltage. The study examine that CE-Jet length of inner liquid is inversely proportional to needle-substrate distance in same time. It was later verified in experimental study by producing stable CE-Jet morphology with 300 μm diameter using optimized parameters (i.e., DC voltage 7.0 kV and inner liquid flow rate 400 nl/min) as compared to other validation studies such as 400 μm and 500 μm. The CE-Jet printing technique investigates significant changes in consistency and stability of CE-Jet morphologies and makes Jet unique and comparable when adjustment accuracy reaches 0.01 mm. PZT sol line structures with a diameter of 1 µm were printed directly on substrate using inner needle (diameter of 120 µm). Therefore, it is considered as a powerful tool for nano constructs production in M/NEMS devices.

## Introduction

Direct writing process in electrohydrodynamic jet (E-Jet) printing^1^ is widely used to form micro/nanostructures on various substrates i.e., silicon wafers, PET and glass^2–4^. Therefore, for high precision manufacturing products in flexible electronic devices, the unconditional controllability of printing process is ensured by stable cone-jet mode during the direct printing process^5^. Many printing methods such as ink jet printing^6,7^, fused deposition modeling and stereo lithography adopt direct writing process to print micro-patterns on highly insulating substrate^8^. However, E-Jet printing is capable of generating micro and nano-scale structures using very simple equipment and large sized printing needles. The main reason behind this phenomenon is that the E-Jet morphology is drawn from the external electric field and not from the pressure applied as in other printing techniques^9–11^. Thus, throughout E-Jet printing process, a high voltage is regulated on the conductive needle system^12^.

At present, coaxial E-Jet printing^13^ is a emergent technology for preparing micro/nanostructures using atomization process for the application in many fields i.e., micro-dispensing, optical devices, cell printing, tissue engineering and sensors^14,15^. The working principle of CE-Jet process is based on use of coaxial needles by regulating flow of inner and outer liquids from the syringe pump. Similarly, CE-Jet morphology obtained during different E-Jet printing modes is significantly influenced by deployed parameters i.e., micro-dripping mode at low flow rate, pulsed jet mode at high printing height, and multi-jet mode at extremely high voltage^16^. Furthermore, the properties (e.g., dynamic viscosity and relative permittivity) of inner and outer solutions affect morphology of cone-jet and stability of CE-Jet printing.

To discuss phenomenal effect of implementing different parameters and modes of E-Jet printing under CE-Jet printing process, we have done literature study to carry out phase field numerical modeling^17^. So far, many researchers have demonstrated CE-Jet printing process such as Sun et al. generated special compositions known as core–shell polymer nanofibers by co-electrospinning method using PEO polymer material as internal fluid and PSU as external fluid^18^. Lee et al. described a multidrug encapsulation technique by coaxial tri-capillary electrospray (ES) system which can synthesize monodisperse PLGA-coated particles containing multiple drugs in one step^19^. Chen et al. studied spraying modes in coaxial jet electrospray with outer driving liquid, and showed that modes of coaxial atomization depend on the physical properties of outer liquid. Hongtao et al. performed coaxial nozzle-assisted E-Jet printing for microscale 3D cell-laden constructs for applications in biomedical fields^20^. Soraya et al. introduced fabrication of micro-nanocapsules by a new electrospraying technique using coaxial E-Jet printing. Hai-liang et al. used coaxial printing method for directly writing stretchable and conductive cable as strain sensor^21^. Ahmad et al. studied generation of multilayer structures for biomedical engineering applications using a new device with three coaxial needles and E-Jet flow^22^. Likewise, Xu et al. performed a finite element simulation of CEA using computational fluid dynamics (CFD) model in Fluent^14^. Similarly, the ability of CFD model to predict output of composite structures was verified by an experimental study^23^. Xiaojun et al. described a coaxial focused electrohydrodynamic jet (CFEJ) printing technique^24^ and printing direct writing nanoscale structures^16^. Therefore, various phase-field studies are reported to be frequently used to improve stability of CE-Jet morphology and control influence of different parameters and properties of inner and outer solutions^17,25–29^. However, currently there are no accurate numerical studies for micro-dripping mode, which significantly help to detect the influence of various parameters on cone profile of inner and outer liquid for application of electronic devices.

To state the limitations of existing work, the present study conducts a particular CE-Jet numerical simulation analysis which addresses influence of different parameters by solving the Navier–Stokes, Cahn–Hilliard and electric field equations during phase field method. The purpose of this simulation work is to illustrate technical routes of direct writing method in CE-Jet printing in order to optimize Jet diameter and print stable continuous line structures using PZT sol and photoresist (AZ703) as inner liquids. In this study, coaxial needle fixture was designed to achieve fixation and adjustment of the inner and outer needles. Based on the leaky dielectric model, coaxial electric jet simulation examines influence of each force on CE-Jet with zero systematic errors by using interface tracking equations during phase field method. Likewise, CE-Jet printing process is considered a tool capable of producing stable and high-resolution microstructures for many applications i.e., MEMS devices, optical devices, cell printing, tissue engineering and sensors etc.

## Three phase field method

### Significance of phase field method

The phase field method has emerged as a powerful and flexible tool for quantitative modeling of coevolution of different microstructures and physical properties at microscale. In phase field method, the microstructure is described by a system of continuous variables and parameters, where microstructure interface has a finite width. The evolution of microstructure is defined in terms of free energy of the system and can be coupled with other physics to provide a complete view of material behavior. Phase field simulation varies from hundreds of nanometers to hundreds of microns and evolves at diffusive time scales. Common applications of phase field method include solidification, phase transformation, irradiation damage, domain evolution in ferro-electric and ferro-magnetic materials, and fluid dynamics. The method is used for three phases of liquids. It is computationally less expensive and practices a Cahn Hilliard equation system. Similarly, classical multiphysics i.e., level set, volume of fluid, or alternative techniques such as particle-based methods and Lattice Boltzmann Method are also volume tracking techniques. These are used for two phases of liquids and computationally more expensive and practice a transport equation system.

In addition, phase field and classical multiphysics approaches are types of volume tracking methods. These methods use different criteria to determine placement of phases in relevant cells. LSM and VOF use a function that describes the smallest distance from interface as a criterion. In first phase, this distance is positive and in next phase is negative. Numerical solutions of advection equation of the function estimate position of the interface. In phase field method, criteria is a function (phase field function) of [− 1, + 1], which in first phase is + 1 and in next part is − 1, whereas zero is interface for this function.

For justification, classical multiphysics methods can be used in boundaries moving either in a predefined or free manner, to describe these boundaries as (defined) 0 levels set of a function. The key is to relate correctly (normal) velocity of moving boundary to evolution of the level set. To avoid moving boundaries and prefer expressing everything in fixed domains, the model used a three phase field method. This is an additional unknown function which is close to 1 in one sub-domain and close to 0 (or 1) in another one—approximating the characteristic function or sign of the level set. The region where phase field changes from value close to 1 to 1 close to 0 (or − 1) is not an interface or a boundary (as in case of level set approach), but a narrow cone. Hence, study concludes phase field approach as one where moving boundary is being diffused. The advantage is obvious; during numerical simulation in a fixed domain identifies moving boundaries as thin regions inside it. However, equation for phase field is compatible with sharp interface model. More precisely, if thickness of transition region approaches 0, which can be controlled by some parameters in phase field model, limit is an interface/boundary moving as in original model. In other words, simulation work ends up with original model involving moving boundaries.

### Materials

The specific materials were selected in simulation model throughout this work. Lead zirconate titanate (PZT) and photoresist (AZ703) were chosen as inner liquid and high viscosity silicone oil (60,000 centistokes) was used as outer liquid. The red-colored photoresist was chosen since it can be easily detected while generating cone profile. The purpose of photoresist material is to be used together with inner liquid and makes it an ideal material. Moreover, it also improves visibility of PZT solution for the examination of CE-Jet morphology. The PZT solution is one of most widely used solution in MEMS devices due to its excellent piezoelectric properties^30,31^. The silicon wafer was used as a substrate for printing purposes with a defined thickness of 0.5 mm. The physical characteristics of inner and outer solutions are given in Table 1.

**Table 1:** Characteristics of functional solutions for CE-Jet printing technique.

Functional sol properties	Density *ρ* (kg·m^-3^)	Dynamic viscosity µ (Pa·s)	Surface tension coefficient σ (N·m^-1^)	Relative permittivity (ɛ)
Photoresist (AZ703)	1050	1.4 × 10^–2^	2.83 × 10^–2^	4.8
PZT solution	1061	1.6 × 10^–2^	1.93 × 10^–2^	22
Silicone oil	976	58.56	22	2.77

### Governing equations of phase field model

**Figure 1:**
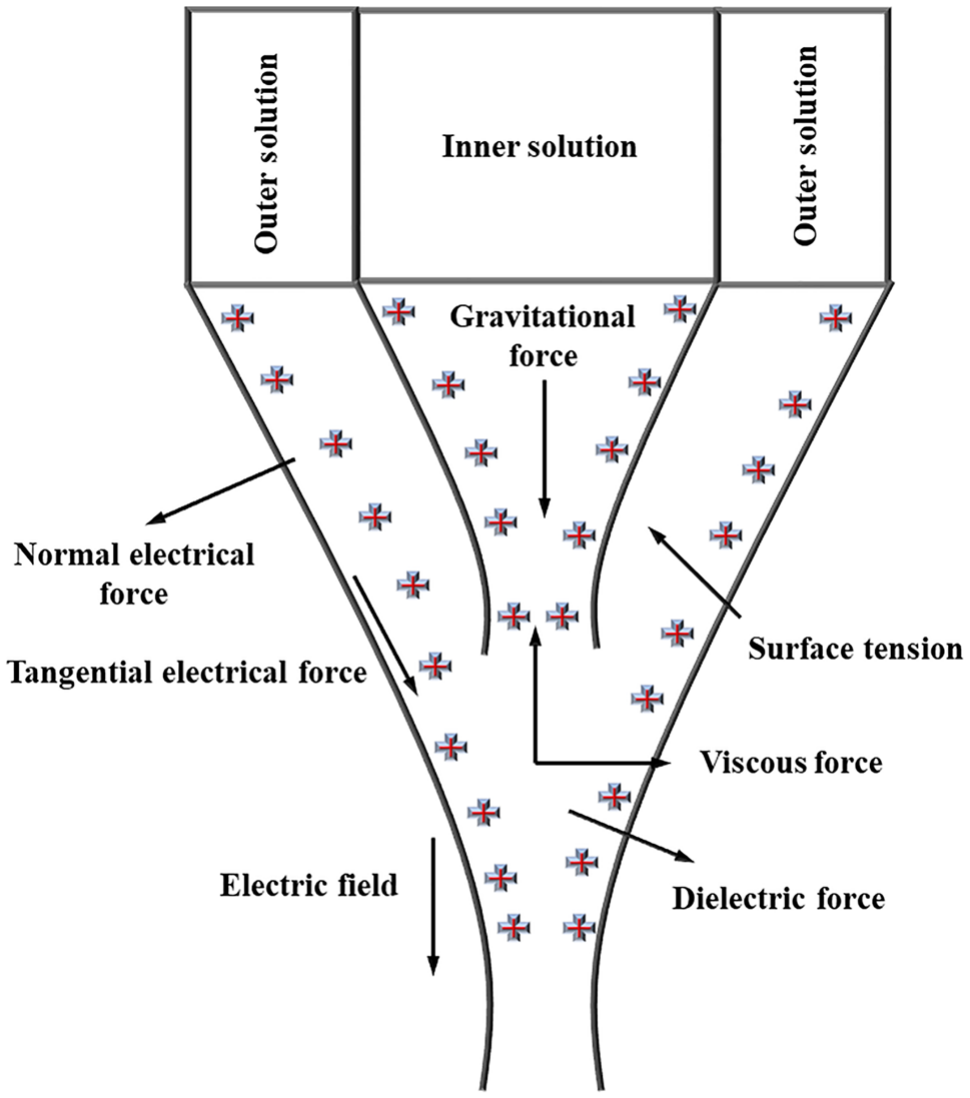
Forces acting around needle tip during CE-Jet printing process.

In this work, three fluids (i.e., inner liquid, outer liquid and air) were assumed to be non-miscible, incompressible and Newtonian and laminar. The three phase flow in phase field contour was based on the leaky dielectric model^32^ and Navier–Stokes–Cahn–Hilliard (NSCH) system^33^. The fluid flow can be defined in following continuity Eq. (1),

1 $$\nabla .\mathop u\limits^{ \to } = 0$$

where $u$ denotes liquid velocity vector of functional solutions. Furthermore, continuity and mass conservation of the Navier–Stokes equation for a three phase system was used to explain forces acting around needle tip during CE-Jet printing process which is shown in Fig. 1. The surface tension defined in momentum equation can be modified as in Eq. (2).

2 $$\begin{aligned} \rho \frac{{\partial \mathop {\left( u \right)}\limits^{ \to } }}{\partial t} + \rho (\mathop u\limits^{ \to } .\nabla )\mathop u\limits^{ \to } =& \nabla .\left[ { - \nabla p\mathop I\limits^{ \to } + \mu (\nabla \mathop u\limits^{ \to } + (\nabla \mathop u\limits^{ \to } ))^{T} } \right] \hfill \\ &+ \nabla .\mathop {\rm T}\limits^{ \to } + F_{st} + F_{es} + F_{P} \hfill \\ \end{aligned}$$

where $\rho$ is liquid density and $pl$ indicates internal pressure of functional solution. Similarly, $\mathop u\limits^{ \to }$ is a dynamic viscosity of the functional fluid, F_st_ is surface tension force of liquid and F_es_ is electric force generated by electric field. The fluid speed with respect to time is described as $\rho \frac{{\partial \mathop {\left( u \right)}\limits^{ \to } }}{\partial t}$ which is important coefficient of interface curve. For the purpose of time constraints, Cahn Hilliard equations were generated over three phase field which were calculated in the software contour^34,35^ as given in Eq. (3)

3 $$\begin{gathered} \left\{ \begin{gathered} \mu .\frac{{\partial \varphi_{i} }}{\partial t} - \nabla .\overrightarrow {\mu } \varphi_{i} = (\nabla .\frac{{{\rm M}_{o} }}{{\sum_{T} }}).\sigma \eta_{i} \hfill \\ \eta_{i} = \frac{{4\sum_{T} }}{\theta }\sum_{i} \prod i \mp j - [\frac{1}{{\sum_{j} }}(\sigma_{i} F(\varphi ) - \sigma_{j} F(\varphi ))][\frac{3}{4} - \sum_{i} (2\varepsilon c\Delta \theta \psi )] \hfill \\ \end{gathered} \right. \hfill \\ i = A,B,C \hfill \\ \end{gathered}$$

where σ expresses surface tension. Moreover, σ and ε are executed independently during Cahn Hilliard equations. M_o_ is a driving force of functional solutions and known as diffusion coefficient. Thus, $\theta$ is a wetting contact angles. The Cahn Hilliard potential is an auxiliary variable which is obtained in variable contour to assist particle directions during the mass flow of liquids as explained in Eq. (4)

4 $$\begin{aligned} F(\varphi ) =& \sigma_{AB} \varphi_{A}^{2} \varphi_{B}^{2} + \sigma_{AC} \varphi_{A}^{2} \varphi_{C}^{2} + \sigma_{BC} \varphi_{B}^{2} \varphi_{C}^{2} \hfill \\ &+ \varphi_{A} \varphi_{B} \varphi_{C} (\sum_{A} \varphi_{A} + \sum_{B} \varphi_{B} + \sum_{C} \varphi_{C} ) \hfill \\ &\sum_{i} = \sigma_{i,j} + \sigma_{i,k} - \sigma_{j,k} \hfill \\ \end{aligned}$$

where A, B, and C are phase variables used to identify location of each phase in the three phase flow model. Further, $\sum_{i}$ denotes total effect of surface tension. The summation of entire three phases with high concentration values is assumed to be equal to unity in each numerical box as given in next Eq. (5).

5 $$\begin{gathered} \frac{3}{{\sum_{i} }} = \frac{1}{{\sum_{A} }} + \frac{1}{{\sum_{B} }} + \frac{1}{{\sum_{C} }} \hfill \\ \varphi_{A} + \varphi_{B} + \varphi_{C} = 1 \hfill \\ \end{gathered}$$

Maxwell tensor equations are used as electrostatic mode in phase field method^36^. The electric field is expressed in the form of electric potential. Hence, by applying Gauss's law to a rectilinear electric medium, it can be reduced to Eq. (6).

6 $$\nabla .(\varepsilon {\rm E}) = \rho_{e}$$

where ε is relative permittivity and $\rho_{e}$ is electric field charge density. Therefore, according to the various studies by Melcher^37^, Saville^38^ and Issa^39^, Eq. (7) is converted from charge conservation equation to Ohmic regime^39^.

7 $$\rho \left(\frac{{\delta \rho_{e} }}{{\delta_{t} }}\right) + \Delta .\rho_{e} u = - \nabla .(\varepsilon {\rm E})$$

Equally when charge distribution and electric field potential are determined, then electric stress can be calculated by Eq. (8).

8 $$F_{st} .\varepsilon = \varphi .(\rho_{e} {\rm E})$$

where E represents chemical potential which is mixing energy generator among auxiliary variables as assumed in Eq. (9).

9 $$F_{e} = \rho_{e} {\rm E} - \left[\frac{1}{2}E^{2} \nabla \varepsilon \right]$$

Similarly, Eq. (9) represents Coulombic force^40^ with all simplifications. This force always acts along electric field which is perpendicular to surface of CE-Jet morphology.

### Physical model

**Figure 2:**
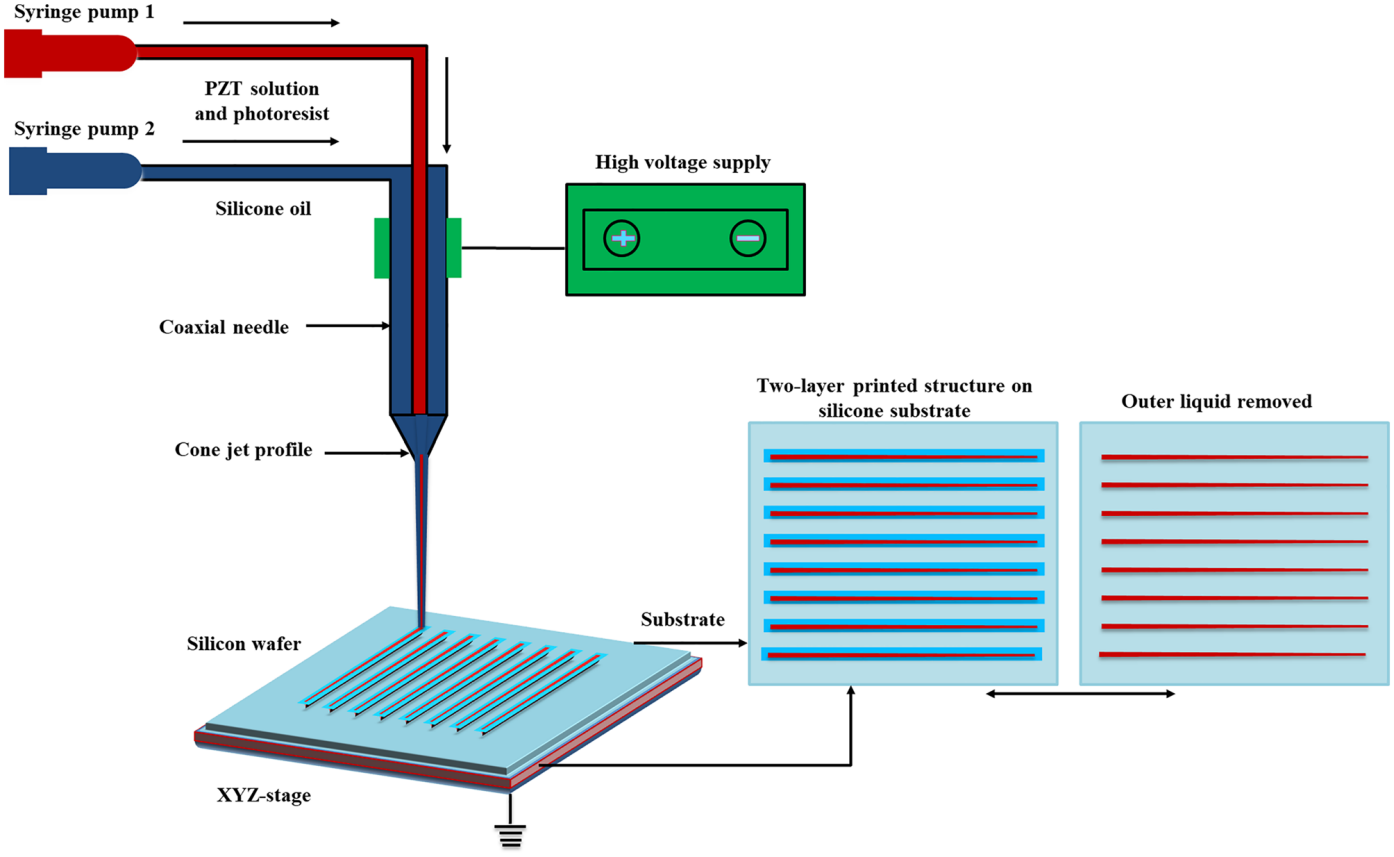
The schematic diagram of the CE-Jet printing system.

**Figure 3:**
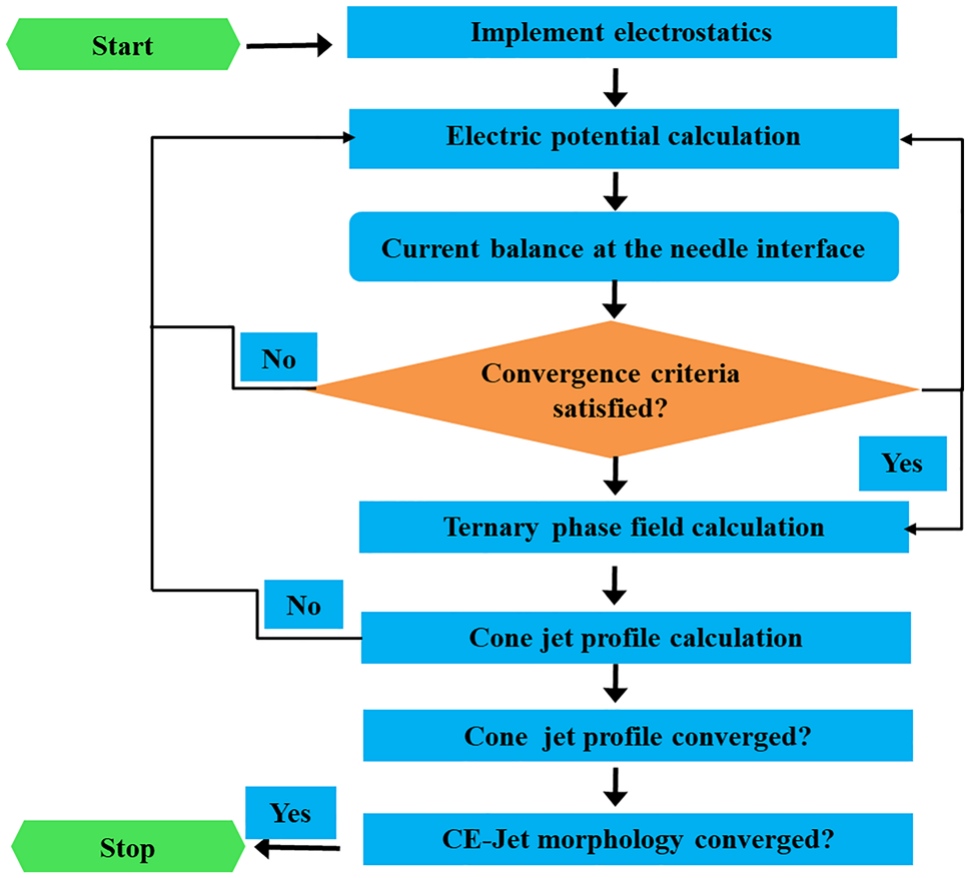
Flow diagram of the phase field modeling.

Subsequently, based on simulation of governing equations, the variables and parameters were used in the phase field method. These variables and parameters were successfully solved in the form of equations to determine electric field for development of cone-jet morphology. Therefore, Maxwell tensor and viscous fluid equations were solved at time dependent solver contour during CE-Jet phase field simulation. The schematic diagram of CE-Jet printing system is illustrated in Fig. 2. Similarly, flow diagram of various computational phases such as A, B and C starts by implementing electrostatic spatial charge density. So, the calculations of electric potential φ(r, z) were kept constant under computational domain of phase flow. The law of conservation of mass and equation of continuity generates body forces in functional fluids that were computed in COMSOL software. Later, electric body force and viscous forces were further added to Navier–Stokes equation to produce three concurrent phases (i.e., two liquid phases and an air phase). The equations were conveniently solved since internal and external solutions were deformed into a CE-Jet morphology due to existence of electric body forces. The ternary phase field of surrounding air was solved to calculate the cone-jet profile. To check extent convergence occurred in the system, iteration was started again from top by calculating electrical body force for cone-jet morphology. Finally, cone-jet profile converged properly during solver configurations and cone-jet shape stopped developing further. The process flow diagram of phase field modeling is shown in Fig. 3.

### Geometric model for numerical simulation

**Figure 4:**
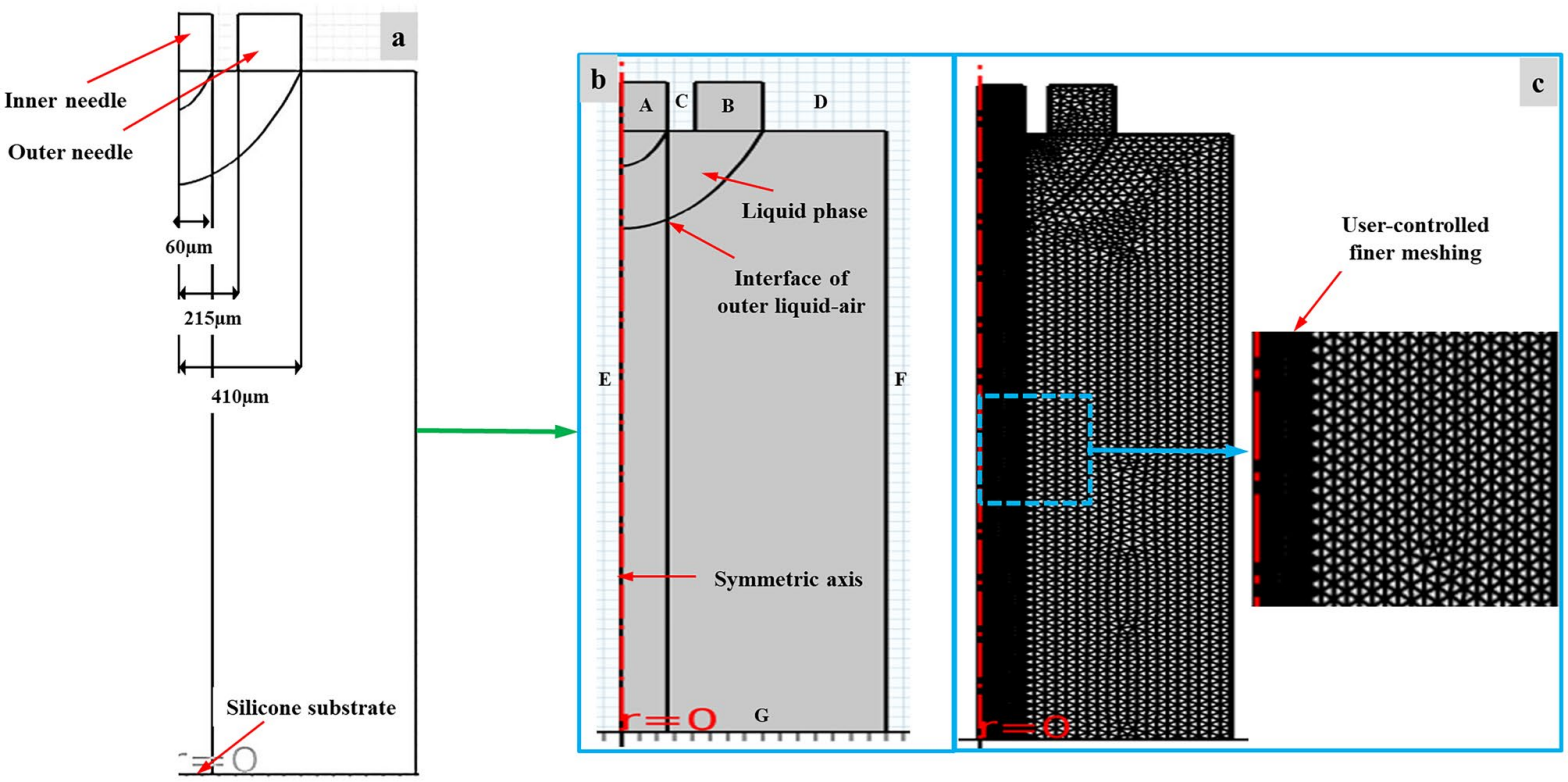
(**a**) The design of 2D sketch (**b**) The axisymmetric geometry of phase field model and particular boundary conditions (**c**) the finer user-controlled meshing for model.

Primarily, 2D axisymmetric sketch was created based on previous studies to construct an accurate model for pursuing appropriate simulation results as shown in Fig. 4a. The axisymmetric geometry and boundary conditions designed in numerical simulation model are displayed in Fig. 4b. Likewise, Fig. 4c illustrates finer user-controlled meshing for simulation model. An axisymmetric geometry was generated to run CE-Jet model under phase field domain. The division of grid is an important part of establishing a simulation model which requires many factors to be considered. The lattice type and quality affects the accuracy and resolution of cone-jet morphology. Therefore, grid division should meet the following requirements:

1. The number of grids directly affects the accuracy of scale calculations and resolution of CE-Jet. Generally, more grid lines then higher the accuracy of calculations and greater the amount of calculation. But when number of grids reaches a certain level, it will not effectively improve calculation accuracy, but will greatly increase amount of calculation.
2. The grid selection was dense and opaque in this work. According to characteristics of the physical model, grids with different densities should be divided into different specific small parts. The advantage of sub-grid is that not only improves the accuracy of solution effectively, but also reduces the amount of calculation.
3. The quality of mesh is mostly restricted by its shape. The quality also affects the accuracy of solution. Poor quality meshes cause the resolution of CE-Jet morphology to not converge properly. A good mesh should have a uniform transition and mesh surface should not be excessively distorted especially at borders and corners. In this paper, a freely divided triangle mesh was used to achieve desired resolution. Considering that formation of CE-Jet morphology is concentrated at exit of the nozzle and axis of symmetry, this article focuses on refinement of mesh to improve the accuracy and effectiveness of simulation resolution. However, times are resolved on the contour in range of (0, 5e−6, 0.5) with respect to time-dependent solver.

The fluid flow and surface tension were described in terms of laminar field flow by implementing ternary phase flow properties. Similarly, coaxial needle system was designed by defining stainless steel material properties in geometry. The applied voltage was just implemented along the walls of needle system instead of functional solution to maintain the simplification of phase field method. In the beginning, when no voltage was applied, the area between needle system and substrate was filled with air density. Here, PZT sol and photoresist (AZ703) were selected as inner liquid and viscous silicon oil was used as outer liquid to conduct simulation experiments for printing substrate. The PZT is sensitive and ultraviolet-cured with following properties, density of 1061 (kg. m^−3^), dynamic viscosity of 1.6 × 10^–2^ µ (Pa s), surface tension coefficient (PZT-Air) of 1.93 × 10–2 σ (N m^−1^), electrical conductivity of 3.1 × 10–4 (s. m^−1^), and relative permittivity of 22. Therefore, during numerical simulation the applied voltage, needle-substrate distance, needle system size, and flow rate was gradually changed while functional solution for printing process was fixed with constant parameters. In this study, voltage was applied within the range of 1 kV to 9.5 kV DC during various experiments. The needle-substrate distance varied from 10 to 18 cm in same time period. Similarly, inner flow rate varied from 100 to 800 nl/min and outer flow rate from 0.5 to 3 µl/min throughout experimental study. Additionally, other parameters with their important implementation values are discussed in detail throughout numerical simulation in results section. The geometric model dimensions simulated in this paper are mainly based on commercial needles, in which the inner diameter of inner needle was 120 µm and outer diameter was 430 µm. Thus, outer needle had an inner diameter of 820 µm and outer diameter was 1000 µm. In order to reduce the amount of calculations and improve simulation efficiency and accuracy, the simulation model has been simplified in following aspects:

1. Converting a three dimensional model into a two-dimensional axisymmetric model.
2. Ignore outer diameter of the outer needle.

The reason for this simplification is that needle shape is axisymmetric and two-dimensional axisymmetric model is sufficient to express needle geometry. The simulation difficulty and calculation amount can be greatly simplified and outer diameter of the needle has no influence on flow field. The boundary circumstances of phase field geometric model are summarized in Table 2. The inner liquid generates inner CE-Jet morphology under high applied voltage and likewise outer silicon oil produced outer CE-Jet under same voltage. Where, φ is initial value of applied voltage, u is fluid initial velocity, and V_0_ is applied voltage to coaxial needle. The voltage was varied from different locations and determined in the form of V during calculation. In addition, Q_inner_ is flow rate of inner liquid and A_inner_ territory represents the cross-sectional area of inner needle. Whereas, Q_outer_ shows flow rate of outer liquid and territory of A_outer_ is difference of cross-sectional area between the inner and outer needle. The CE-Jet morphology mainly depends on the velocity of external fluid and distribution of electric field in phase field method. Therefore, due to strong effect and variance in electric field potential, flow field of various functional solutions transformed exclusively in region around the axis of symmetry. In this situation, meshes located in region approaching the axis of symmetry were refined by applying user-controlled sequence type. Thus, it can also help to improve the efficiency and accuracy of Taylor cone profile morphology.

**Table 2:** The boundary conditions of the phase field method.

Boundary	Electrostatic field	Hydrodynamic field
A: Inner needle inlet	φ = V_0_	u = Q_inner_/A_inner_
B: Outer needle inlet	φ = V_0_	u = Q_outer_/A_outer_
C: Wall of inner needle	φ = V_0_	u = 0
D: Wall of outer needle	φ = V_0_	u = 0
E: axisymmetric space	φ_r_ = 0	u_r_ = 0
F: Boundary of computational territory	φ = V	P = 0
G: Outlet of coaxial needle	φ = 0	P = 0

## Results and discussion

### The simulation analysis of coaxial electric Jet

**Figure 5:**
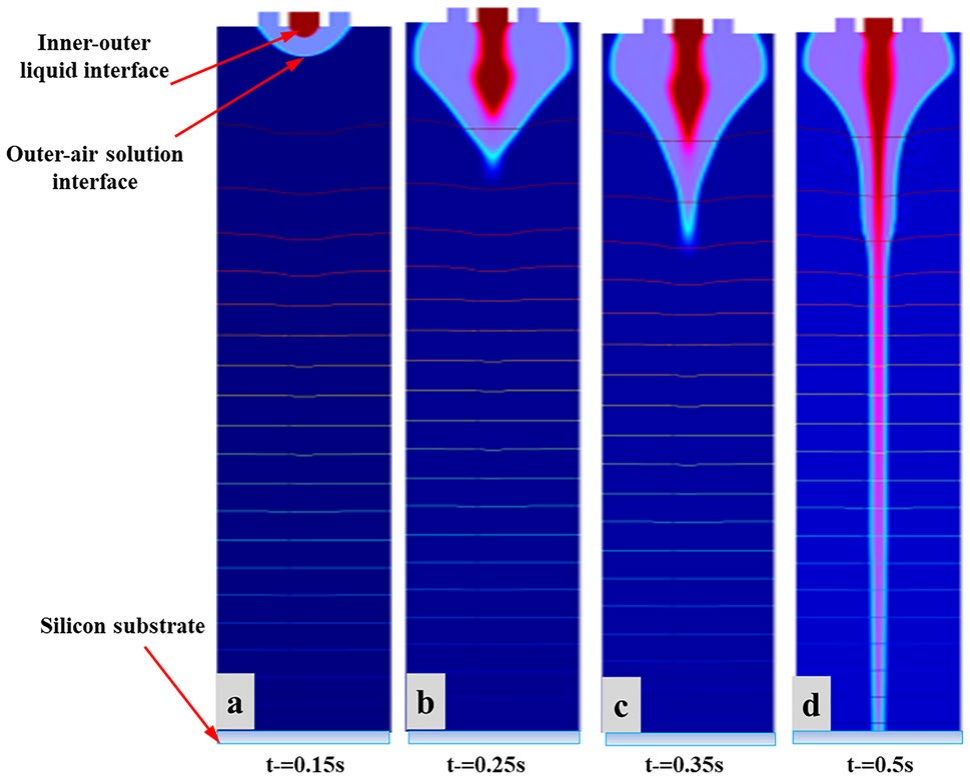
The specific changes of ternary phase field morphologies (**a**–**d**) at different time steps (0–0.5 s) with applying electric field.

The simulation results are mainly divided into two parts specifically, change of CE-Jet morphology when no electric field is applied to needle and when electric field is applied to the needle. Thus, comparing the difference between two situations, law of electric fields impact on functional fluid was found to be significant. Figure 5a shows three phase flow at different time intervals (0, 5e−5, 0.5 s) when no electric field was applied. In case of engraving, it can be seen that liquid was in needle when subjected only to fluid dynamics such as gravity, surface tension and viscous force. Surface tension and contact angles are two major parameters describing a meniscus shape and it depends on the intermolecular forces interacting between two phases. The functional liquid gradually accumulates on aperture since flow rate of external liquid is larger than that of inner liquid. Therefore, a dual hemispherical structure is finally formed in which outer fluid wraps the inner fluid.

Figure 5b–d illustrates the change in three phase flow at different time intervals when an electric field was regulated. It can be observed that under gravity, the counter amongst combined action of surface tension, viscous force and electric field force, the pulling action of CE-Jet morphology at exit of needle was gradually reduced by half. The spherical shape evolved into a stable cone-jet profile. It can be comprehended that electric field strength predominates in formation of CE-Jet. The space charge density at different time intervals gradually increased after the application of electric field around interface. According to Melcher leaky dielectric model and theory proposed by Taylor^41,42^, there is a free charge in solution under influence of applied electric field that track interface around needle tip. In addition, due to internal fluid the relative dielectric constant is greater than external fluid, so internal fluid induces more charge than the external fluid. The parameters used in entire process during the CE-Jet printing technique are given in Table 3**.**

**Table 3:** Parameters used in the CE-Jet printing technique.

Parameters	Voltage (kV)	Flow rate (nl/min)	Dynamic viscosity (Pa*s)	Relative permittivity	Printing height (cm)
1	5.5	300	3.5	150	10
2	6.0	400	3.0	100	12
3	6.5	500	2.5	50	14
4	7.0	600	2.0	40	16

### The influence of applied voltage on CE-Jet morphology

**Figure 6:**
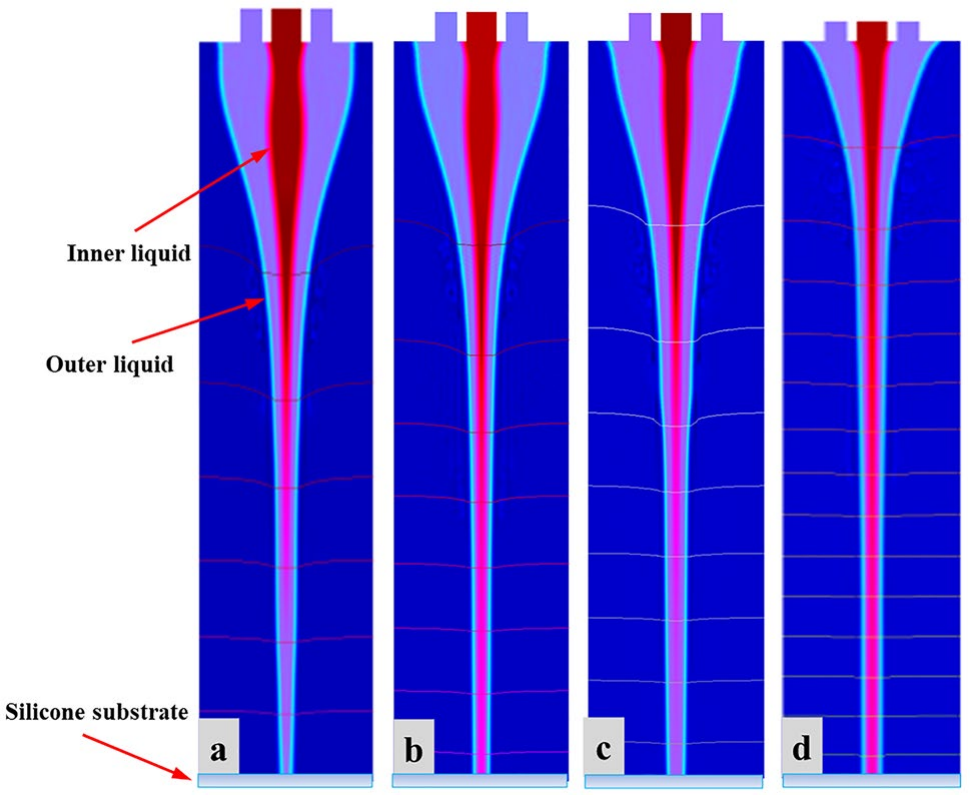
The specific morphologies of the simulated CE-Jet at different applied dc voltages (**a**) 5.5 kV (**b**) 6.0 kV (**c**) 6.5 kV and (**d**) 7.0 kV.

In order to determine the effect of applied voltage on CE-Jet morphology, the flow rate and needle-substrate distance were kept constant during numerical simulations by regulating internal fluid flow rate as 400 nl/min, external liquid flow rate as 3 µl/min and needle-substrate distance as 10 cm. The different simulation experiments were performed while applied voltage was set to 5.5 kV, 6.0 kV, 6.5 kV, and 7.0 kV. The charge mobility is reduced significantly in high viscosity liquids which causes reduction in conductivity. So, the high range of voltage may lead to a stable cone-jet structure and effect of solid surfaces can be taken into account for stability. It can be seen from Fig. 6a–d that as the voltage increases, the Taylor cone as well as CE-Jet diameter becomes smaller; hence the size of CE-Jet is inversely proportional to applied voltage. This is mainly due to increase in electric potential which causes the tangential electric field to act strongly on cone-jet profile. The evolution at interface gets bigger dragging CE-Jet morphology towards transition and thinner zone, which produces smaller diameter of inner liquid CE-Jet. During simulation when voltage is too low, the electric field produces weak electrical forces, which are insufficient to overcome the surface tension and viscous force.

**Figure 7:**
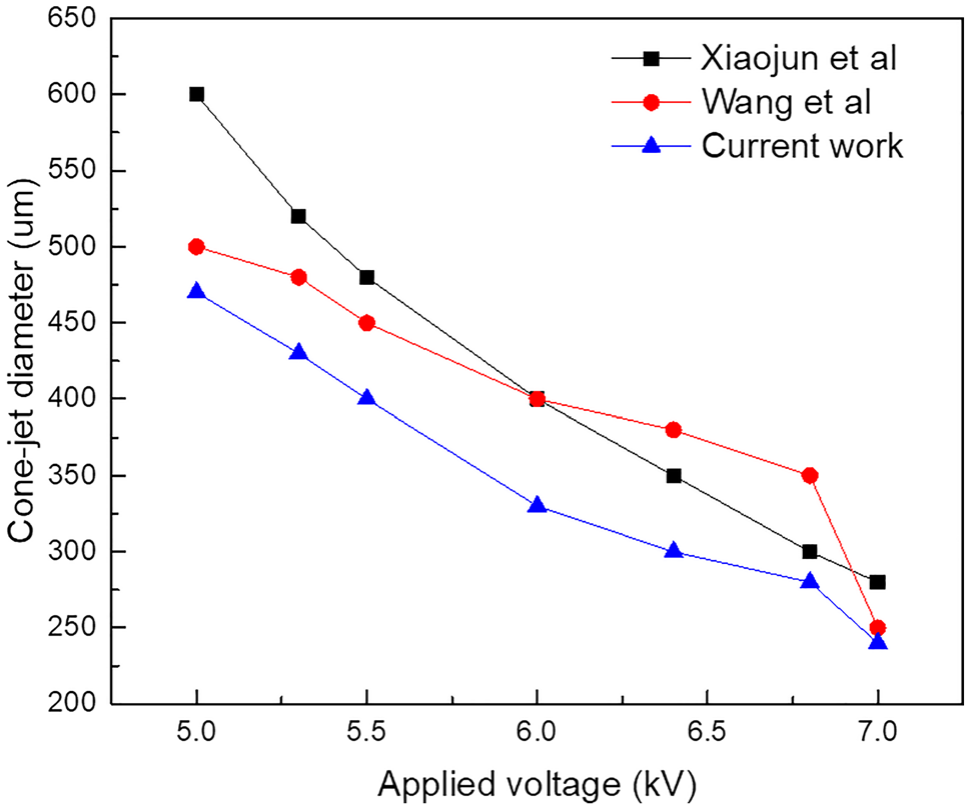
The particular changes during validation of results under the voltage applied with other simulation models.

Subsequently, it accumulates evenly in the functional fluid at needle outlet in larger quantities than fluid sprayed on the substrate and CE-Jet formed at this time appears very coarse. So far, theoretically when value of voltage is adjusted, it causes a greater tangential electric field force resulting in a smaller CE-Jet diameter. Furthermore, it is expected to produce various printed microstructures with smaller size. However, when the voltage is too high (7.0 kV) and liquid sprayed to the substrate is more as compare to outer liquid that can be supplied by the syringe pump, the outer liquid will break in needle tip and form an empty space. Subsequently, as the voltage increases further (more than 7.0 kV), the instability of multiple jets and even electric field breakdown may occur during phase field method. To verify results obtained from influencing parameters in the existing research model we compared the applied voltage values with some similar research works^13,16,28^. So, in this simulation work, applied voltage of 7.0 kV provides a subtle form of internal CE-Jet morphology. Thus, Fig. 7 displays diameter of CE-Jet as a function of voltage applied to PZT and silicon oil, respectively. It is clearly seen that as voltage increases, the diameter of CE-Jet gradually decreases. So, if the diameter of CE-Jet increases, this will cause to generate bigger microdroplet size on substrate as simulation experiments proves. Further, CE-Jet morphology shows that results of phase field method have sufficient accuracy in terms of diameter and at high voltage values relationship between applied parameters are highly correlated. This indicates that at low voltage values the difference is < 5%. This occurs due to fast decay of residual charges on the substrate and its quick electrically induced effect. The comparison of printed cone-jet diameter to different optimized parameters obtained during CE-Jet printing technique is given in Table 4

**Table 4:** Comparison of results at different optimized parameters during CE-Jet printing technique.

Validation work	Voltage (kV)	Inner solution flow rate (nl/min)	Outer solution flow rate (µl/min)	Needle-substrate distance (cm)	Needle size (µm)	CE-Jet diameter (µm)
Current work	7.0	400	3	10	120	300
Xiaojun et al	6.5	200	2	16	130	400
Wang et al	6.5	250	2.2	15	130	500

### The influence of flow rate on CE-Jet morphology

**Figure 8:**
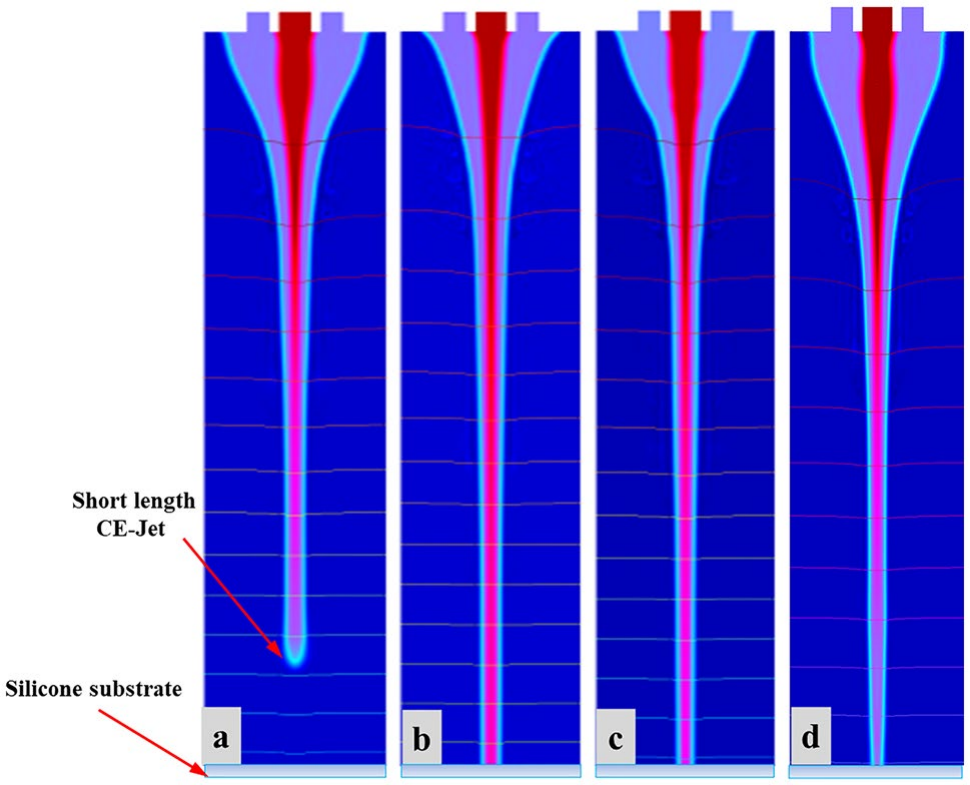
The specific morphologies of the simulated CE-Jet at different flow rates of inner solution (**a**) 300 nl/min (**b**) 400 nl/min (**c**) 500 nl/min and (**d**) 600 nl/min.

Based on the phase field technique, we maintain applied voltage and needle-substrate distance constant to investigate the effect of internal fluid flow on CE-Jet diameter by setting the voltage at 7.0 kV, external liquid flow rate at 3 µl/min and needle-substrate distance at 10 cm. Different simulation experiments were performed with flow rate values set at 300 nl/min, 400 nl/min, 500 nl/min, and 600 nl/min. It can be seen from Fig. 8a–d that as flow rate increases, the Taylor cone becomes longer and CE-Jet diameter changes significantly. Initially the flow rate value set at 300 nl/min indicates a smaller cone-jet diameter but CE-Jet did not reach the substrate surface due to effect of low electric field around needle tip. Therefore, phase field simulation indicates direct relationship of CE-Jet size to the internal fluid flow rate. This situation is mainly due to the increase in robust transportation of liquid molecules, which is caused by unit time. The amount of functional liquid accumulates at the end of needle that has ability to increase speed of ternary phase during phase B variable. Moreover, when flow rate is too high (600 nl/min), the liquid accumulated at needle outlet is too much inundated and it forms cluster. Thus, the cluster of molecules causes cone-jet profile to be unstable in the experiments and increases diameter of CE-Jet as shown in Fig. 8d. Hypothetically, it has been proved that if internal liquid flow rate value is smaller, then the CE-Jet diameter develops smaller. Similarly, further printed microstructure size is also produced smaller on the substrate surface. However, the internal fluid flow rate is limited by minimum flux of liquid molecules that is provided by syringe pump. So far, when flow is too small, then fluid supplied by the syringe pump is unable to maintain stability of the Taylor cone and provide the liquid needed for coaxial jetting. It is concluded that width of generated cone-jet profile increases with enhancement of flow rate of functional solution.

**Figure 9:**
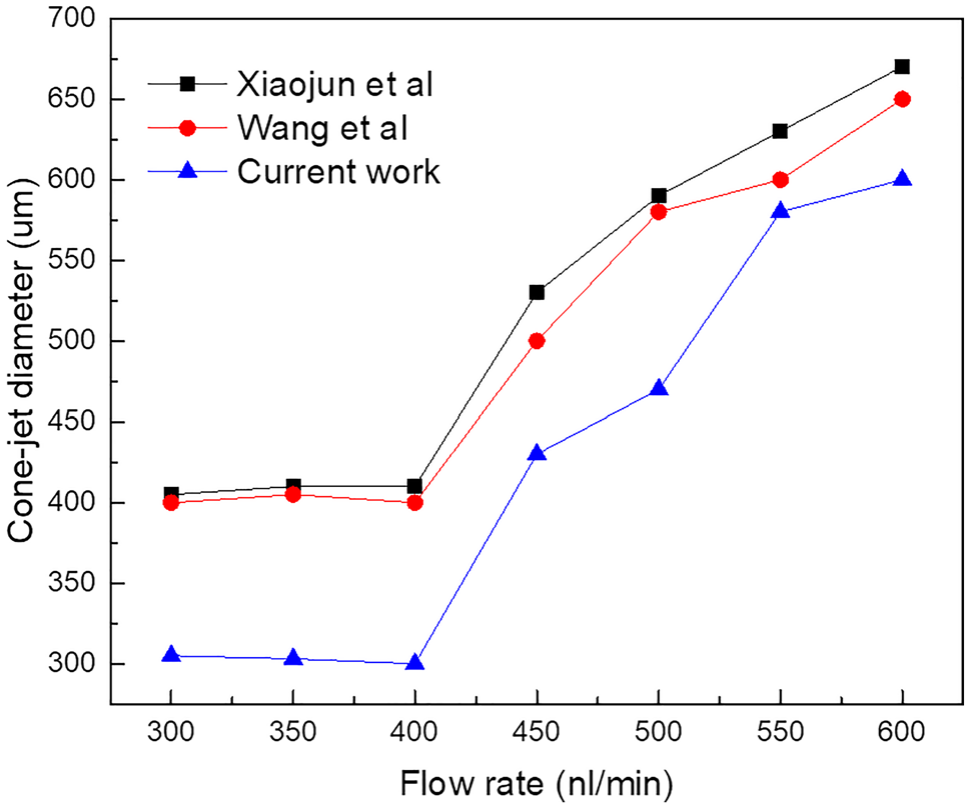
The specific changes when verifying results under flow rate regulation with other simulation models.

During particular simulation experiments in this paper, the flow rate value of 400 nl/min was determined to be optimal providing a subtle form of internal cone-jet morphology compared to verification results. Furthermore, Fig. 9 demonstrates specific changes when verifying results under flow rate regulation with other simulation models. It has been observed that functional liquid properties have a remarkable effect on diameter of CE-Jet. In this effort, the CE-Jet diameter and electric current have been quantified as a function of the flow rate. However, the CE-Jet current increases with an increase in electric potential due to the generation of higher flow velocities and charges in the CE-Jet. In addition, the calculated total current for silicone oil is obviously larger than PZT due to its much higher conductivity. As flow rate increases, the diameter of CE-Jet also increases because conductivity of a PZT solution is smaller than silicon oil. The effect of external electric field on PZT solution is smaller, therefore, the diameter of outer liquid cone-jet is found to be larger than inner solution cone-jet morphology.

### The influence of dynamic viscosity on CE-Jet morphology

**Figure 10:**
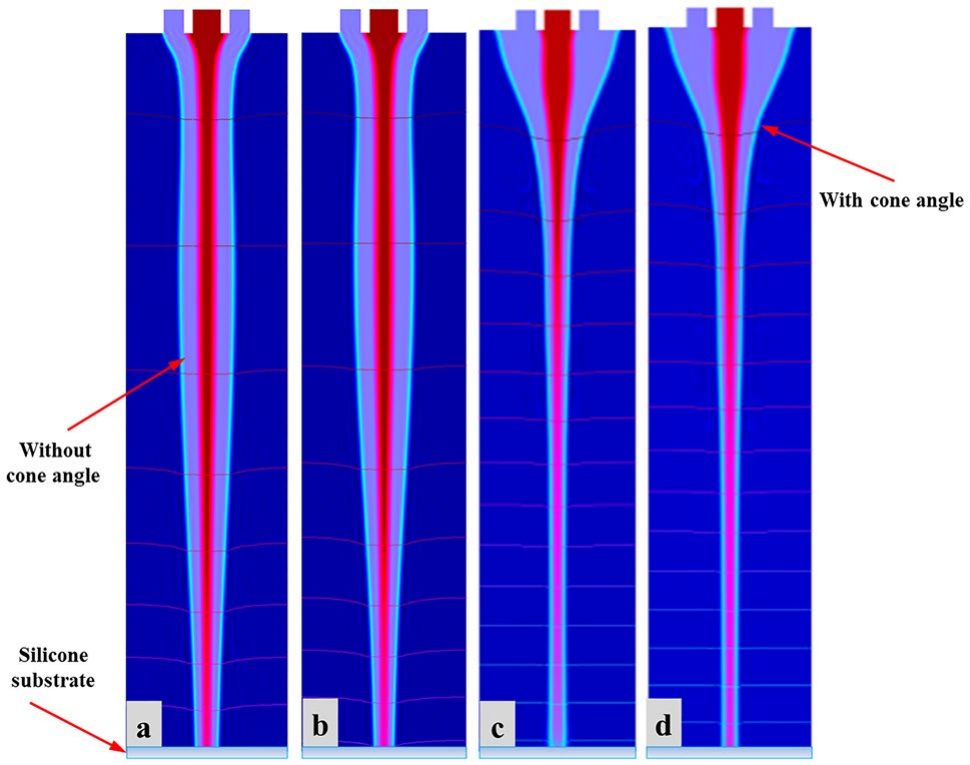
The specific morphologies of the simulated CE-Jet at different dynamic viscosity of outer solution (**a**) 3.5 Pa * s (**b**) 3.0 Pa * s (**c**) 2.5 Pa * s and (**d**) 2.0 Pa * s.

PZT is an important piezoelectric material with high dielectric constant, high relative dielectric constant and high electromechanical coupling. This article uses PZT sol as internal liquid and high viscous silicon oil as outer liquid. In this simulation model dynamic viscosity of silicon oil was varied at different levels to investigate the influence of molecules concentration on CE-Jet morphology. Keeping the flow rate, applied voltage and printing height constant during the numerical simulation, the influence of dynamic viscosity was determined on the CE-Jet morphology. Initially during the experiment, it was found that concentration of silicon oil molecules was too high. This causes needle to clog, so silicon oil was used in this simulation with a concentration of 3.5 Pa*s for printing stable cone-jet shape. In order to obtain printed microstructure with smallest size, according to simulation results in Fig. 10a, b the outer solution with dynamic viscosity of 3.5 Pa*s and 3.0 Pa*s was applied using syringe pump. The simulation results showed that if outer liquid is more viscous, then cone-jet shape is unstable. The bulging impact appeared in middle of CE-Jet morphology which blew outer liquid due to weak effect of electric field on the terminal sweep unit. Furthermore, as dynamic viscosity of outer solution reduces, the bulging effect on CE-Jet morphology disappears and proper cone-jet profile evolved during simulation experiments. Similarly, results in Fig. 10c, d demonstrate that when viscosity was set at 2.5 Pa*s, the outer solution generates proper cone-jet shape due to strong electric field around needle tip, where concentration of silicon oil was swift according to fluid properties. In addition, when dynamic viscosity was very low (2.0 Pa*s), functional liquid did not accumulate at needle outlet and passes through needle interface under the strong effect of electric field without agglomeration. Thus, bunch of molecules made a stable cone-jet profile during the experiments and decreases diameter of CE-Jet as shown in Fig. 10c. Hypothetically, it has been proved that if dynamic viscosity value is kept smaller, then CE-Jet diameter decreases over time. Similarly, further size of printed microstructure can be produced smaller on substrate surface for the application of electronic devices.

### The influence of relative permittivity on CE-Jet morphology

**Figure 11:**
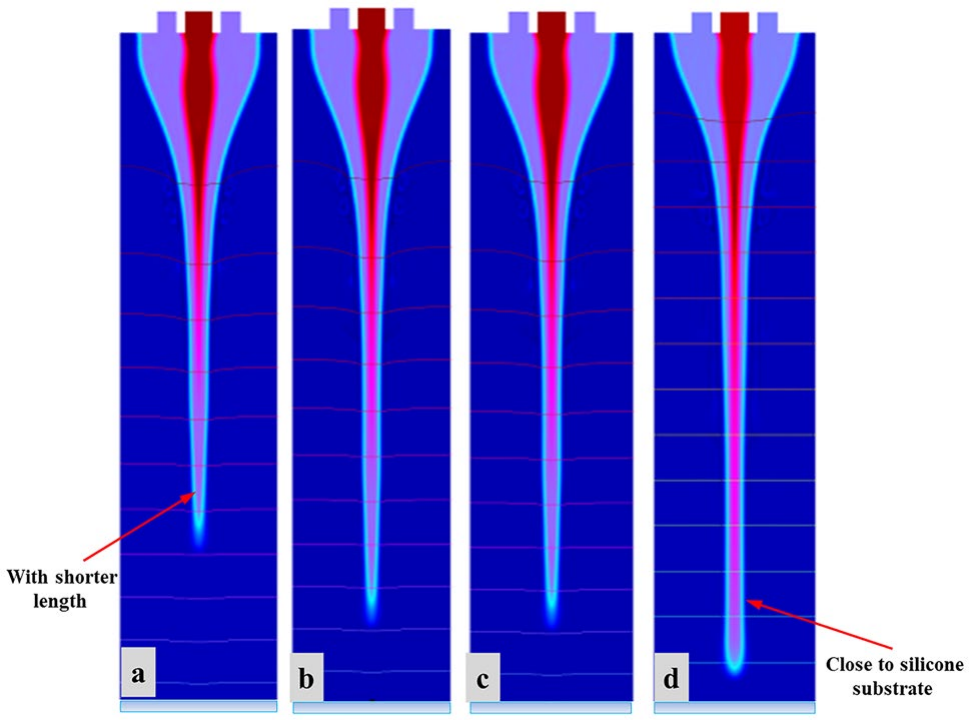
The specific morphologies of the simulated CE-Jet at different relative permittivity of outer solution (**a**) 150 (**b**) 100 (**c**) 50 and (**d**) 40.

At present, there are several researches on parametric evaluation, which describe stability of coaxial focusing electric jet printing technology based on double needles. As a core factor in printing experiment, relative permittivity plays a key role in improving accuracy of micro-nano structures. In this study, we determine that relative permittivity has immense structural impacts on CE-Jet morphology. A well-designed needle system should have certain advantages such as good reliability, easy processing, inter-changeability, simple assembly and disassembly and adjustable coaxiality to check proper influence of relative permittivity. The existing needle device guarantees that coaxiality predominantly depends on the processing. Throughout phase field method, constant values of relative permittivity of outer liquid and air were maintained as 2000 and 10, respectively. Likewise, the permittivity of outer solution was adjusted to check the influence on needle interface. As presented in Fig. 11a, permittivity of inner liquid was set 150 indicating that CE-Jet was shorter and did not reach substrate surface due to influence of high permittivity of outer solution. However, as the permittivity of inner liquid decreases (100 to 40) in simulation experiments, length of CE-Jet increases and approaches to substrate surface as exhibited in Fig. 11b–d. Thus, relative permittivity also influences both solutions inside needle system, which generates appropriate cone-jet profile around needle tip. These shortcomings in length of CE-Jet will inevitably affect printing quality and accuracy, as well as increase the time durations.

### The influence of needle-substrate distance on CE-Jet morphology

**Figure 12:**
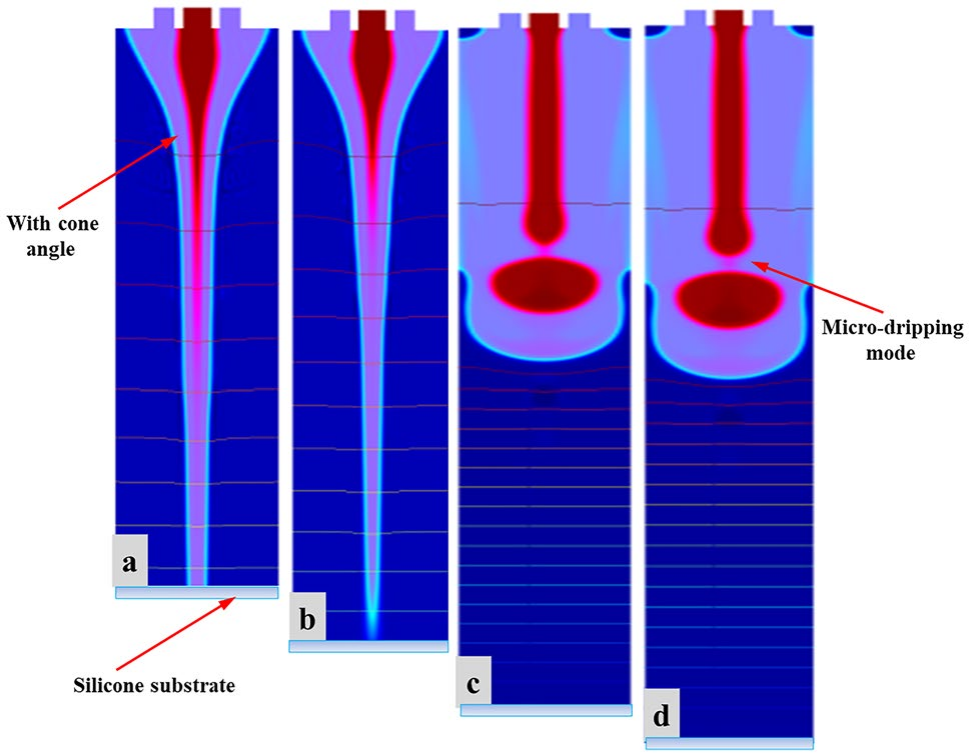
The simulation results of CE-Jet morphology with different coaxial needle-ground electrode distance at the same time (**a**) 10 cm (**b**) 12 cm (**c**) 14 cm and (**d**) 16 cm.

Figure 12 shows corresponding CE-Jet morphology at the same time when needle-substrate spacing is 10 cm, 12 cm, 14 cm, and 16 cm, respectively. The remaining process parameter settings in four simulations remain unchanged, where applied voltage is 7.0 kV, the inner liquid flow rate is 400 nl/min, and the outer liquid flow rate is 3 µl/min. Figure 12a–d shows as needle-substrate distance increases, CE-Jet formation becomes slower. According to analysis of variable-speed motion of CE-Jet and Eq. (10), the length of CE-Jet in time (t) can be expressed as.

10 $$L = \frac{{t^{2} }}{2}\left( {F_{\mu } } \right. + \rho Qgt + \frac{{2q_{v} V}}{{r_{c} \ln (4H/r_{c} )}}$$

It can be seen from Eq. (10) that CE-Jet length is inversely proportional to the needle-substrate distance in same time. Because by increasing needle-substrate distance under same voltage, the spatial electric field intensity decreases and the charge density and electric field force at two-phase interface decrease. Thus, it produces micro-dripping mode which as a result generates micro-drops. Similarly, at the same time as CE-Jet formation speed slows down then CE-Jet length is reduced. Further, when the voltage is constant and needle- substrate distance exceeds a certain value due to low electric field strength in space, then electric field force at the two-phase interface is not sufficient to overcome surface tension of liquid and CE-Jet will not be formed. In this simulation, spraying when needle- substrate distance exceeds to 16 cm, CE-Jet cannot be formed and transform into multi-jet phenomenon. Therefore, by increasing the needle-substrate distance more voltage is required i.e. to maintain electric field. More, on CE-jet morphology the effects on needle-substrate distance will be addressed with more applied voltage. However, needle-substrate distances more influence on the CE-Jet stability rather that cone-jet morphology.

### The influence of needle size on CE-Jet morphology

**Figure 13:**
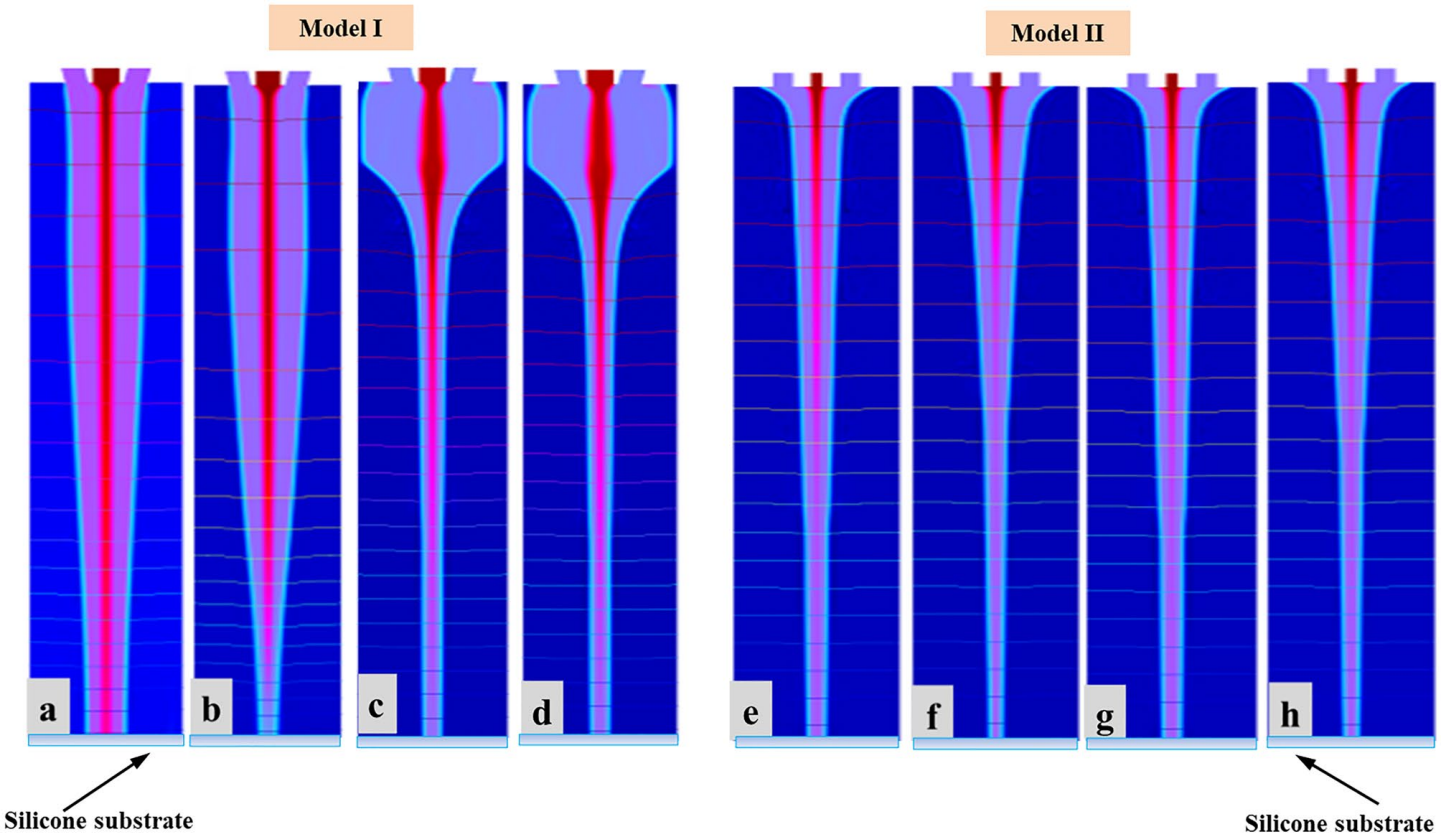
The different simulation results at different needle diameter and shape: Model (I) shows different simulation morphologies at different time intervals (**a**–**d**) by changing shape and size of outer needle: Model (II) shows different simulation morphologies at different time intervals (**e**–**h**) by changing size of inner needle.

Different simulation results were obtained by changing inner (110 µm) and outer (810 µm) diameter of needle to examine stability of CE-Jet morphology. The purpose of different models was to practice a phase field method to control CE-Jet (cone jet) profile by designing different structures of coaxial needles. Correspondingly, coaxial needle was used in simulation model by modifying with different diameter and size; later different CE-Jet morphologies were obtained by applying different constant parameters (i.e., voltage 7.0 kV, inner flow rate 400 nl/min, external flow rate 3 µl/min, printing height 10 cm). While, maintaining all parameters constant, the CE-Jet morphologies at applied voltage (7.0 kV), inner flow rate (400 nl/min) and external flow rate (3 µl/min) were evaluated under stable conditions, as presented in Fig. 13 at different time durations. The model (I) describes that in the beginning when constant values were implemented over the time intervals (t = 0, 5e−5, 0.5 s), CE-Jet evolved towards substrate surface resulting in smaller size due to opposite diameter of needles. However, over time, CE-Jet diameter of outer liquid increased in middle zone over the last time (t = 0.35 s) as it reached surface of silicon substrate. The outer diameter increases due to accumulation of abundant residual charges and weak electric field around needle interface. The simulation results in Fig. 13a, b deals with bulging shape of outer CE-Jet but at time intervals (t = 0.4 s) and (t = 0.45 s), the outer liquid evolves as an appropriate cone-jet shape as shown in Fig. 13c, d. The cone-jet was formed near needle tip due to thick streamline of electric field. The initial phase movement of outer liquid towards setting time to generate developed CE-Jet morphology at time interval (t = 0.5 s) to reduce bulging effect. It produces stable inner CE-Jet, where the CE-Jet morphology was fully in developed condition.

Similarly, in model (II) inner diameter of needle was changed and keeping the same dimensions of outer needle, the inner CE-Jet was obtained smaller in size as compared to CE-Jet of outer liquid. The inner liquid produces clot inside needle apex due to smaller diameter and liquid molecules size stuck inside needle because of needle structural changes and liquid viscous properties. Therefore, it can be seen from model (II) in Fig. 13e, f that CE-Jet is very short in length obtained at time intervals (t = 0.35 s) and (t = 0.4 s) respectively. Furthermore, as time increases, the morphology of inner CE-Jet shortened and liquids dispersed due to unstable behavior of PZT solution. The effect of electric field was very uniform on outside region of needle as shown in Fig. 13g, h. Finally, it was concluded that effect of needle size and change in diameter influences CE-Jet morphologies when functional liquid reaches substrate surface at time interval (t = 0.5 s).

## Discussion

From the simulation results, achieved morphology of CE-Jet was stable by using optimized parameters. Similarly, to verify this conclusion, coaxial electric jet printing experiments were performed based on optimized simulation parameters. The needle size and parameters were kept constant (inner flow rate 400 nl/min, outer liquid flow 3ul/min, needle-substrate distance 10 cm) by adjusting applied voltage to 7.0 kV. The physical properties of silicon substrate do not scatter charge vectors of functional solutions on the substrate surface and among the locations of needle-substrate; therefore jetting phenomenon becomes very stable. This paper initially prepared a PZT sol accordingly to match properties of silicone substrate. Because silicon wafers also known as a slice or substrate is a thin slice of crystalline silicon. It’s primarily used as substrate for integrated circuits (ICs) since silicon is considered a highly stable semiconductor. Similarly, a silicon wafer has the best possible surface finish and micro-roughness on the order of less than 10A haze free angle. Thus, 3.55 g of titanium isopropoxide (Titanium (IV) isopropoxide) mix with 5.39 g of zirconium (IV) propoxide and magnetically stir for 5 min; then add 5 ml Glacial acetic acid, 10 ml 1-propanol and 9.95 g lead acetate trihydrate, magnetically stirred at 70 °C until the lead acetate trihydrate is completely dissolved. Lastly, add 11 ml of 1-propanol and 10 ml of glacial acetic acid, and stir on a magnetic stirrer for 4 h. A PZT sol with a concentration of 0.6 mol/L was obtained because PZT is an important piezoelectric functional material for M/NEMS devices. During the printing process, a two-layer structure is obtained and the printed bilayer structure can be heated at up to 300 °C for 30 min to accelerate the evaporation of organic solution and to solidify printed bilayer structure from substrate. This heating process can also be used to reinforce the bonding strength between inner structure and silicone substrate, which can prevent the structures washing away in removing process of outer solution. Finally, the silicone substrate will be placed in isopropyl alcohol to remove outer encapsulated material until the substrate has cooled to room temperature. The microscale structures only composed of inner material on the substrate can be obtained after removing residual liquid by heating the microstructures at temperatures (50–100 °C) for 15 min.

**Figure 14:**
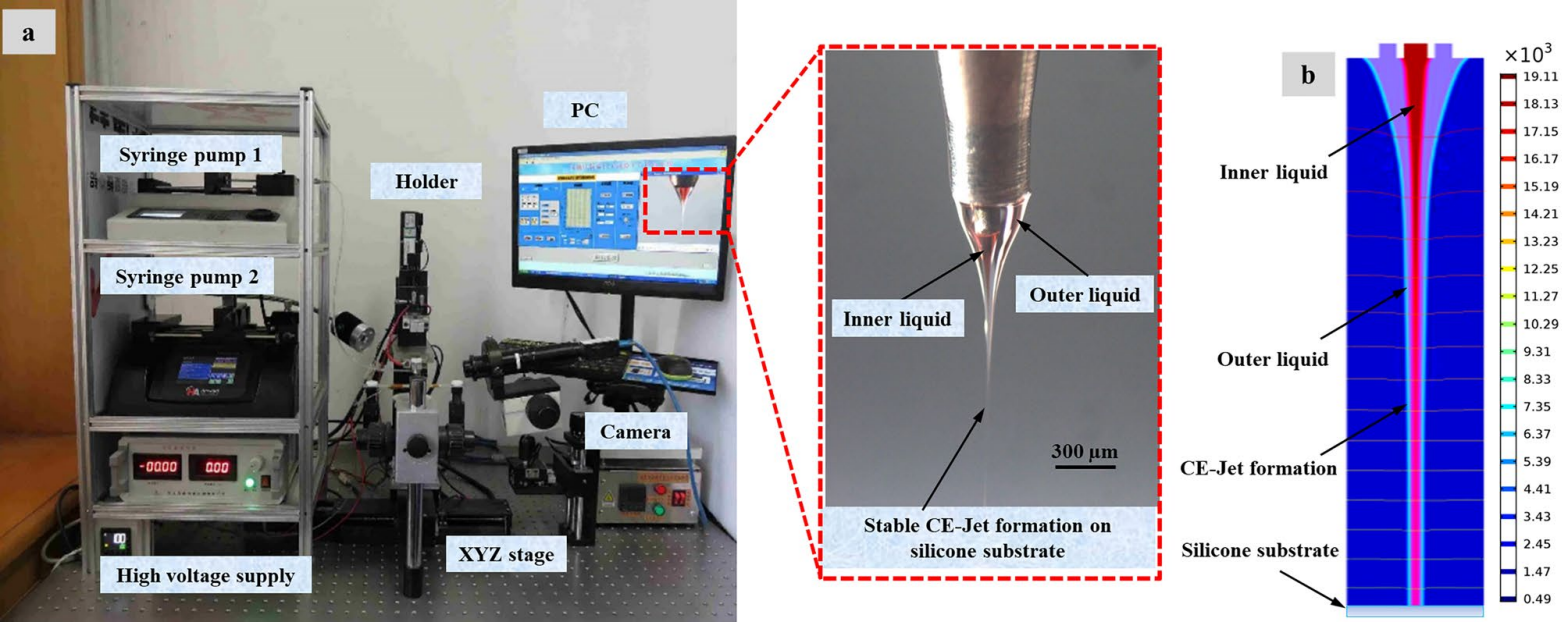
The photos of CE-Jet and the microstructure of photoresist at certain printing parameters: (**a**) experimental photo (**b**) simulation result.

According to Fig. 14b, the formation of coaxial electric jet is mainly affected by electric field force, surface tension, viscous force, gravity, etc. The combined effects of forces are attributed to three aspects of force including gravity, electric field force, and fluid dynamics in 2D axisymmetric geometry. Therefore, it is necessary to introduce four physics Fields, i.e., gravity fields, electrostatic fields, flow fields, and multiphysics that require coupling. Where, the gravity field is included in flow field, the gravitational acceleration is set to 9.8 m s^−2^ and the direction is Y. Because the dielectric constant of material jumps across the interface field, so study need to customize the relative dielectric constant and space charge density in electrostatic field. Therefore, 2D axisymmetric geometry in simulation shows the complete description of model for each parameter and variable used in this work.

**Figure 15:**
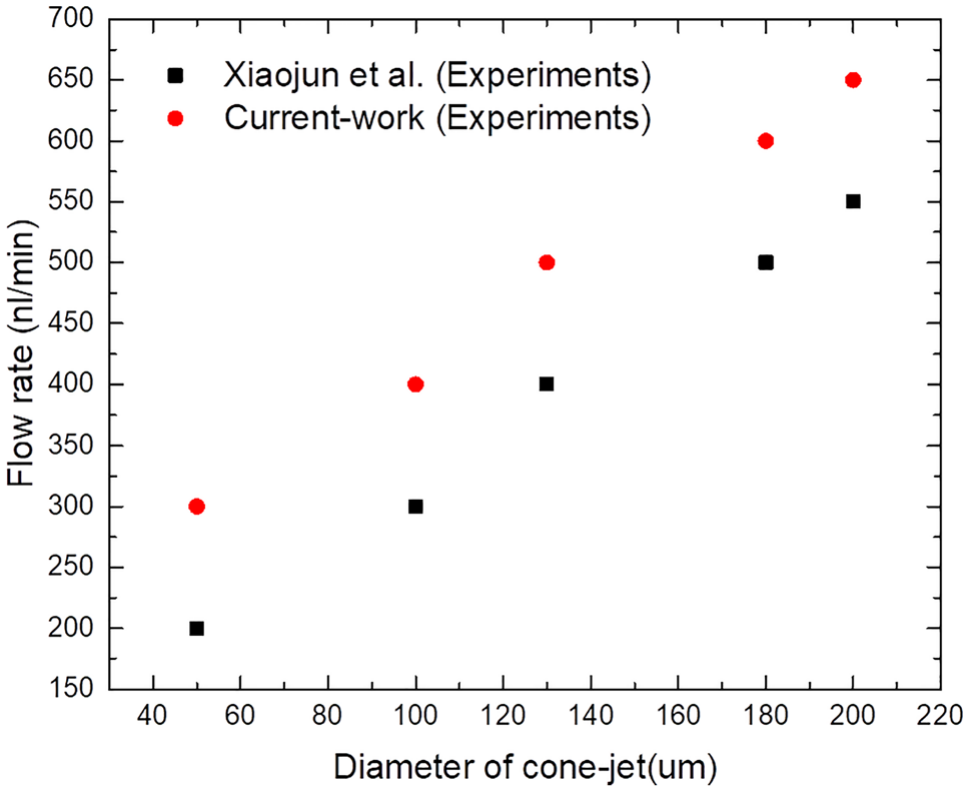
The CE-Jet diameter as a function of the flow rate for inner PZT solution.

As can be seen from the photos of CE-Jet and microstructure of photoresist at certain printing parameters in Fig. 14a, the Taylor cone and CE-Jet diameter get smaller; therefore this rule is consistent with simulation results. In addition, Fig. 14a depicts the creditability and correctness of simulated CE-Jet for the application of MEMS devices. Figure 15 demonstrates that phase field model results are in good relationship with experimental results and are optimized by Xiaojun et al.^13^ because this model use small values of voltage and inner flow rate to generate the CE-Jet morphology. The electric potential did not resolve governing equations properly and a simplified model is assumed throughout the study.

**Figure 16:**
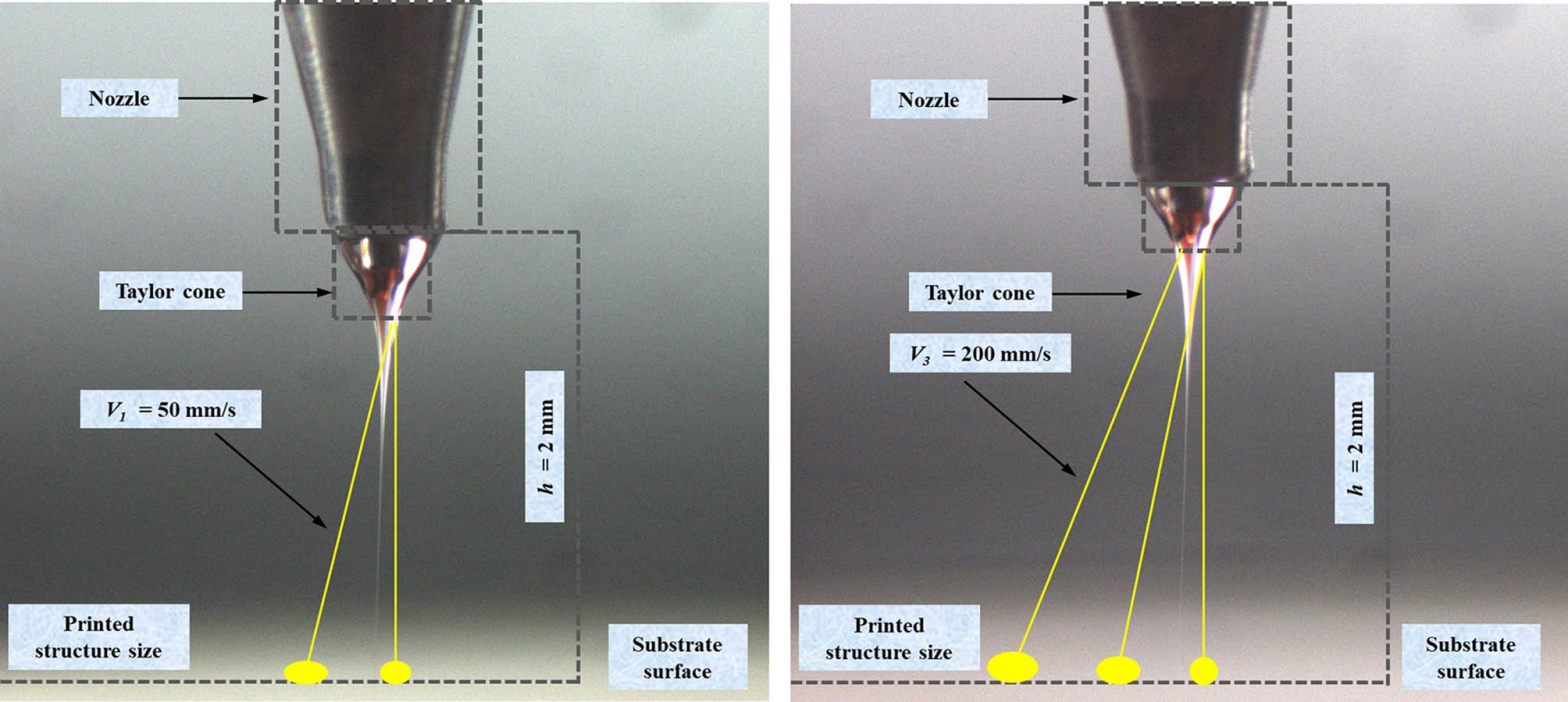
Relation of the substrate speed and the size of printed structures.

**Figure 17:**
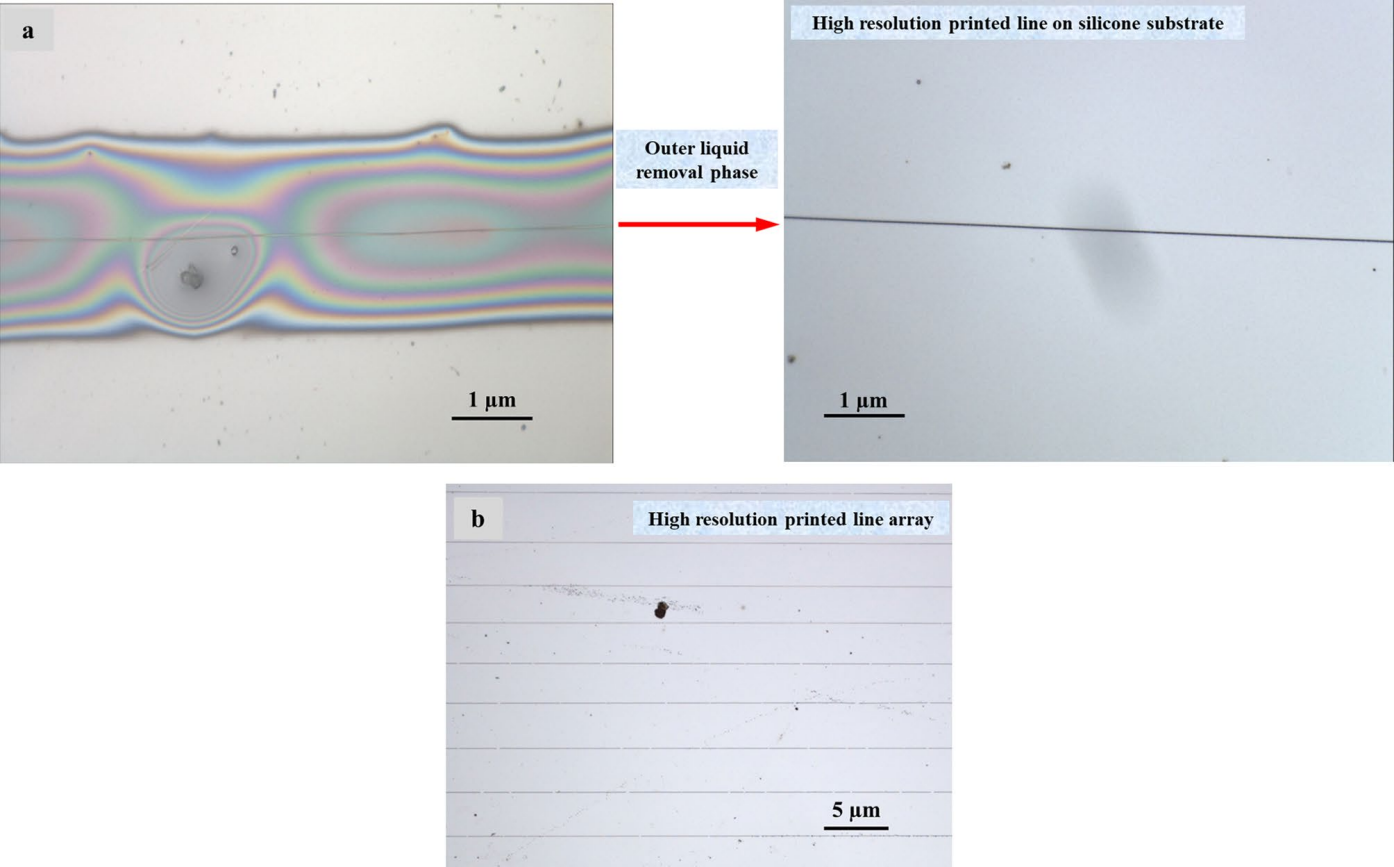
(**a**) The two-layer structure printed using CE-Jet with photoresist as the inner ink and silicone oil as the outer solution and (**b**) the printed line array.

Figure 16 displays the change trend graph of printing structure size with the substrate speed. This indicates that size of printing structure decreases with increasing substrate speed. When the substrate speed is adjusted from 50 to 400 mm∙s^−1^, the print structure size is reduced from 10 to 5 µm. Stable CE-Jet is a continuous fluid flow and CE-Jet between the materials is printed on substrate because the outer layer of liquid is a highly viscous solution. The movement of Jet between the needle and substrate is mechanically dragged (see Fig. 16). As the substrate speed increases, then mechanical drag force increases and the Jet is drawn more finely. At the same time, increasing substrate speed will also enhance the stability of Jet and improve the orderliness of printing structure. Figure 17a, b presents two-layer line and array structure printed using CE-Jet with photoresist as the inner ink and silicone oil as outer solution.

In order to verify influence of substrate structure on morphology of printed line structure, we printed and fabricated ordered PZT line arrays on a patterned substrate as shown in Fig. 17b. The substrate is a single crystal silicon wafer and patterned structure is prepared by CE-Jet printing. After the printing is completed then adhesive film and PZT line structures on the surface are removed together to obtain an ordered line structure as shown in Fig. 17a. The successful printing of this structure shows that coaxial electro-fluid jet printing process is not affected by morphology of silicon substrate in the manufacture of special structures and has high substrate adaptability.

## Conclusions

In this work, the basic theory for CE-Jet printing technology is presented which is based on phase field simulation. The influence of the key printing parameters on Taylor cone morphology is studied by numerical simulation. The needle-substrate distance and relative diameter of needle is designed on the basis of tracking interface. Therefore, simulation results display that correlation between applied voltages and diameters of CE-Jet morphologies is very strong. The correlation between needle-substrate distance and printing conditions was explained by electric field, Navier–Stokes equation and Maxwell pressure tensor. To conclude, the transformation of physical model of CE-Jet to geometric model determined that CE-Jet simulation results and E-Jet theory are in good relationship which indicates the correctness of proposed phase field method. The numerical simulation experiment produced optimized parameters which are then used in experimental study to generate a stable CE-Jet profile. Therefore, it was used to produce linear microstructures that would assist in printing of electronic and M/NEMS devices. Likewise, based on this simulation model and results, an adjustable coaxial needle can be easily designed and manufactured for printing various micro-nano structures. To prove simulation parameters the coaxial printing experiment equipment was used to print PZT continuous line structures with the size of 1 µm on silicone substrate. The parameters and printing results conclude that three phase field method is decent and most capable compared to other methods in literature studies for governing stability of Taylor cone morphology in CE-Jet printing technique.

## References

[1] Lee, Y. Direct Printing and Electrical Characterization of Conductive Micro-Silver Tracks by Alternating Current-Pulse Modulated Electrohydrodynamic Jet Printing. **139**, 1–10 (2017).

[2] Barton K (2010). Mechatronics technical note a desktop electrohydrodynamic jet printing system. Mechatronics.

[3] Byun, D., Nguyen, V. D., Dutta, P. & Park, H. C. A Hybrid Inkjet Printer Utilizing Electrohydrodynamic Jetting and Piezoelectric Actuation. (2010). doi:10.1143/JJAP.49.060216

[4] Qin, H., Wei, C., Dong, J. & Lee, Y. AC-pulse modulated electrohydrodynamic ( EHD ) direct printing of conductive micro silver tracks for micro-manufacturing AC-Pulse Modulated Electrohydrodynamic ( EHD ) Direct Printing of Conductive Micro Silver Tracks for Micro-Manufacturing. (2014). doi:10.14809/faim.2014.0763

[5] Yu, L., Tse, L. & Arbor, A. Airflow assisted electrohydrodynamic jet printing: an advanced micro-additive manufacturing technique. 1–8 (2016).

[6] Riheen MA, Saha TK, Sekhar PK (2019). Inkjet printing on PET substrate. J. Electrochem. Soc..

[7] Yin, Z. P., Huang, Y. A., Bu, N. Bin, Wang, X. M. & Xiong, Y. L. Inkjet printing for flexible electronics: Materials, processes and equipments. *Chin. Sci. Bull.* **55**, 3383–3407 (2010).

[8] Characterization of flexible temperature sensor fabricated through drop-on-demand electrohydrodynamics patterning.pdf.

[9] Tse, L., Barton, K., Tse, L. & Barton, K. Airflow assisted printhead for high-resolution electrohydrodynamic jet printing onto non-conductive and tilted surfaces Airflow assisted printhead for high-resolution electrohydrodynamic jet printing onto non-conductive and tilted surfaces. **054103**, 1–6 (2015).

[10] Search, H., Journals, C., Contact, A., Iopscience, M. & Address, I. P. Controlled deposition of nanoparticle clusters by electrohydrodynamic atomization. **1519**, (2004).

[11] Han Y, Dong J (2016). Design of integrated ring extractor for high resolution electrohydrodynamic (EHD ) 3D printing. Procedia Manuf..

[12] Choi J (2008). Drop-on-demand printing of conductive ink by electrostatic field induced inkjet head. Appl. Phys. Lett..

[13] Zhao X (2019). Numerical simulation of coaxial electrohydrodynamic jet and printing nanoscale structures. Microsyst. Technol..

[14] Xu Q (2013). Coaxial electrohydrodynamic atomization process for production of polymeric composite microspheres. Chem. Eng. Sci..

[15] Xu S, Poirier G, Yao N (2012). Fabrication and piezoelectric property of PMN-PT nanofibers. Nano Energy.

[16] Wang D (2018). Nanoscale coaxial focused electrohydrodynamic jet printing. Nanoscale.

[17] Kim J (2012). Phase-fieldmodels formulti-component fluid flows. Commun. Comput. Phys..

[18] Sun Z, Zussman E, Yarin AL, Wendorff JH, Greiner A (2003). Compound core-shell polymer nanofibers by co-electrospinning. Adv. Mater..

[19] Lee YH, Bai MY, Chen DR (2011). Multidrug encapsulation by coaxial tri-capillary electrospray. Colloids Surf., B.

[20] Liang H, He J, Chang J, Zhang B, Li D (2018). Coaxial nozzle-assisted electrohydrodynamic printing for microscale 3D cell-laden constructs. Int. J. Bioprinting.

[21] Yan HL (2016). Coaxial printing method for directly writing stretchable cable as strain sensor. Appl. Phys. Lett..

[22] Ahmad Z (2008). Generation of multilayered structures for biomedical applications using a novel tri-needle coaxial device and electrohydrodynamic flow. J. R. Soc. Interface.

[23] Yan F, Farouk B, Ko F (2003). Numerical modeling of an electrostatically driven liquid meniscus in the cone-jet mode. J. Aerosol Sci..

[24] Zhao X (2019). Numerical simulation of coaxial electrohydrodynamic jet and printing nanoscale structures. Microsyst. Technol..

[25] Wei, W. *et al.* Numerical simulation of the cone-jet formation and current generation in electrostatic spray - Modeling as regards space charged droplet effect. *J. Micromech. Microeng.* **23**, (2013).

[26] Kim, J. & Lowengrub, J. Phase field modeling and simulation of three-phase flows.pdf. *Interfaces Free Bound.* **7**, 435–466 (2005).

[27] Herrada, M. A., Vega, E. J., Montanero, J. M. & Popinet, S. Numerical simulation of electrospray in the cone-jet mode. **026305**, 1–8 (2012). 10.1103/PhysRevE.86.026305 23005852

[28] Rahmanpour M, Ebrahimi R (2016). Numerical simulation of electrohydrodynamic spray with stable Taylor cone–jet. Heat Mass Transf/Waerme- und Stoffuebertragung.

[29] Boyer F (2002). A theoretical and numerical model for the study of incompressible mixture flows. Comput. Fluids.

[30] Nguyen TD (2012). Piezoelectric nanoribbons for monitoring cellular deformations. Nat. Nanotechnol..

[31] Guo R (2000). Origin of the high piezoelectric response in PbZr_1__−__x_TixO_3_. Phys. Rev. Lett..

[32] Schnitzer O, Yariv E (2015). The Taylor-Melcher leaky dielectric model as a macroscale electrokinetic description. J. Fluid Mech..

[33] Saville DA (1997). ELECTROHYDRODYNAMICS: the Taylor-Melcher Leaky dielectric model. Annu. Rev. Fluid Mech..

[34] Achtzehn T, Müller R, Duft D, Leisner T (2005). The Coulomb instability of charged microdroplets: dynamics and scaling. Eur. Phys. J. D.

[35] Gomez A, Bingham D, De Juan L, Tang K (1998). Production of protein nanoparticles by electrospray drying. J. Aerosol Sci..

[36] Tang K, Gomez A (1994). Generation by electrospray of monodisperse water droplets for targeted drug delivery by inhalation. J. Aerosol Sci..

[37] Of, R. & Shear, I. Electrohydrodynamics: a review of the role of interfacial shear stresses.

[38] Rajabi, A., Javadi, E., Rahman, S., Sereshkeh, P. & Morad, M. R. Experimental characterization of an extended electrohydrodynamic cone-jet with a hemispherical nozzle hemispherical nozzle. (2018). doi:10.1063/1.5037991

[39] Ashrafizadeh A, Nikfar M (2016). On the numerical solution of generalized convection heat transfer problems via the method of proper closure equations – part II: application to test problems. Numer Heat Transf Part A: Appl.

[40] Yang GH, Mun F, Kim GH (2016). Direct electrospinning writing for producing 3D hybrid constructs consisting of microfibers and macro-struts for tissue engineering. Chem. Eng. J..

[41] Abbas, Z. *et al.* Numerical simulation of electrohydrodynamic jet and printing micro- structures on flexible substrate. *Microsyst. Technol.* **1**, (2020).

[42] Abbas, Z. *et al.* Microelectronic Engineering Numerical simulation of stable electrohydrodynamic cone-jet formation and printing on flexible substrate. *Microelectron. Eng.* **237**, 111496 (2021).

